# A theoretical review on the interplay among EFL teachers’ professional identity, agency, and positioning

**DOI:** 10.1016/j.heliyon.2023.e15510

**Published:** 2023-04-18

**Authors:** Xiaolin Li

**Affiliations:** School of English, Jilin International Studies University, Changchun, Jilin 130117, China

**Keywords:** Professional identity, Agency, Positioning, EFL Teacher, Language education

## Abstract

Language teaching is by nature a complex and social practice with numerous personal and contextual aspects. It hinges upon how teachers perceive themselves, the way they are perceived by others, and their roles and positioning in the classroom and its occurring interactions. Despite the proliferation of research on English as a foreign language (EFL) teachers' professional identity, its association to agency and positioning has been widely ignored in academia. Urged by this backdrop, the current study was a bid to theoretically explore the relationship among teachers’ professional identity, agency, and positioning in language education. To do so, the theoretical and empirical underpinnings of this line of research are reviewed referring to different definitions, dimensions, approaches, and conceptualizations of each construct. Additionally, to approve their strong linkage, scientific findings from previous studies are built upon. Furthermore, the implications of this line of inquiry for different stakeholders in EFL contexts, especially teachers are presented in detail. Finally, the study enumerates a number of research gaps in this domain and suggests some future directions for enthusiastic scholars.

## 1 Introduction

Teachers are core players in education and the classroom [1]. Their emotions, practices, identities, and competencies influence different aspects of teaching and learning processes [[2], [3], [4]]. As a ubiquitous factor shaping teachers' self-image and instruction, identity has been the focus of numerous studies over the past decades [[5], [6], [7]]. It is the sense, self-image, and self-awareness that an individual has of him/herself which is projected personally or perceived by others [8]. The identities that teachers craft in a context, significantly affect their work commitment, engagement, efficacy, and professional norm adherence [9]. This signifies that identity is dynamic, multiple, context-specific, relational, and socially constructed [10,11]. Likewise, teacher identity as a complex phenomenon has been approved to be ever-changing, situational, and non-linear [12]. Due to its multiplicity, discontinuity, and social nature, teacher identity is still a slippery and murky concept in education, especially when it is examined in association with how it affects teachers' pedagogical practices [10]. It can be shaped and reshaped through time and in interactions in professional contexts and communities of practices (COPs). As teacher identity is socially made in groups, the negotiations and dialogues between the self and other members vehemently (re)constructs EFL teachers’ professional identity or how they see themselves as professional teachers.

Teachers usually establish different identities based on the roles that they take in COPs. Hence, their identity is mutable, non-imposed, and the outcome of an interplay of personal, professional, and discoursal dimensions [13]. As a less researched venue for teachers' professional identity development, *agency* refers to one's capacity to act decisively and reflectively in his/her world [14,15]. Similarly, Vähäsantanen [16] defined professional agency as having the power to act, influence issues, and make decisions/choices concerning one's professional identities and career. Moreover, Hitlin and Elder [17] considered agency as a core concept that manifests a person's power and influences life's structured pathways. Like identity, teachers' agency is dynamic, relational, dialogic, and social-oriented [18]. Depending on the context and roles taken by a teacher, his/her level of agency changes. So, no EFL teacher is completely an independent agent in the classroom nor is he/she controlled totally by external factors [19]. As pinpointed by Teng and Bui [20], teacher's agency is a pivotal precondition and element for his/her professional identity formation and reformation. Teachers with agency normally take initiatives and actions in their professional setting and reflect on their practices to pedagogically grow [21]. They are no longer simple members of a community or just being in a context; instead, they are active participants in a context who utilize different personal and contextual resources to act in a specific way or cause a change [22].

This idea is in stark contrast with traditional approaches to teacher education in which syllabi, curricula, policies, and practices were all prescriptive and representative of the dominant accountability and exam-oriented atmosphere that de-professionalized teachers and ruined their agency [23]. Now, scholars call for a performance-oriented educational culture that cares for EFL teachers' agency at personal, structural, and cultural levels that improve education over time [24]. Owing to their dialogical, interaction-based, and social essence, both teachers' professional identity and agency are affected by a critical variable called *positioning*. Obviously, the way teachers position themselves and their students in the classroom has a considerable impact on their identity construction and level of agency [[25], [26], [27], [28]]. The concept of positioning grew out of *positioning theory* as a framework to investigate classroom interactions and their impacts on learning and identity [27]. It clarifies how potential actions can be restricted or reinforced by one's positions in a professional context. The notion of *positioning* refers to dialogically situating oneself or others with specific rights and obligations [29]. In EFL contexts, as teachers agentively assign themselves and others particular positions, they (re)construct their professional identity, as well. By positioning, EFL teachers can obtain or lose their right/agency to act or even talk in a certain way. Hence, it can be argued that teachers' professional identity, agency, and positioning are in a reciprocal and circular relationship each affecting the others. Although some studies have been conducted on these variables separately, their linkage in EFL contexts has mostly been kept under the carpet. Against this yawning gap, the present review study intended to offer a theoretical analysis of the interplay among EFL teachers' professional identity, agency, and positioning, which has remained under-researched, to date. In doing so, it explains the concepts, their associations, related theories and studies, research gaps and future trends, and implications of this line of inquiry.

## 2 Literature review

### 2.1 Identity and teacher identity

The concept of *identity* refers to the general self-image and awareness that individuals craft in relation to “who they are” and “what roles they take” in a socio-cultural context [30]. It is a dynamic and evolving attribute that is both personal and social in that its (re)construction is due to a network of internal and external factors and processes [31,32]. It is essential to note that one's identity is context-specific in the sense that a person can develop various identities in light of different professional contexts and entrusted roles. Consequently, an individual can craft multiple identities in relation to his/her family, profession, culture, policy, and religion [33,34]. People usually negotiate their identities in their interactions with other community/discourse members by accepting, reinforcing, downplaying, and challenging identity features and behavioral/thinking patterns [22]. Despite the booming interest in demystifying learner and teacher identity in different educational contexts, the very concept of ‘identity’ is still poorly defined [35]. It also lacks an analytical framework that effectively serves researchers in different disciplines [36]. In academia, mostly, the personal dimension of identity has been explored and the sociological side has been ignored [37].

The growing body of research on identity in education has provided fresh insights into the concept of *teacher identity*, which refers to a self-image or what it means to be a teacher [38]. In simple terms, it concerns how a teacher perceives him/herself as a teacher [38]. It is a concept whose development and (re)construction is essentially social and interaction-oriented. It has personal and social dimensions. The personal dimension concerns one's image created by previous experience as a learner and a classroom teacher, while the social dimension is about the roles that a teacher takes at work. The notion of teacher identity is hard to be exactly conceptualized due to its *multiplicity*, *discontinuity*, and *social nature* [10]. These traits signify that the concept is dynamic, negotiated, dialogical, transitional, ongoing, relational, enacted, and evolving over time [22,39,40]. These concerns reflect a movement form linguistic identity toward language teacher identity, which has gained momentum over the past two decades [41]. Teacher identity has been substantiated to reside and situate in community of practice [42] that is influenced by one's ideologies and discourses in the environment [2,43]. In an influential study on teacher identity, Yazan [42] contended that professional identity is positionally constructed through the “mediation of micro-interactional and macro-structural transactions via meso-level activity” (p. 40). He ran multiple case studies and argued that teachers' identity negotiation is highly correlated to their positioning. Likewise, Trent [44] maintained that EFL teachers construct their identity depending on how they discursively position themselves in the context. In sum, to comprehensively capture teachers' identity, researchers are recommended to take into account all these complexities and dimensions of being, becoming, and acting as a teacher.

#### 2.1.1 Approaches to teacher identity

Throughout the burgeoning history of identity and teacher identity, different perspectives have been proposed each capitalizing on a specific aspect of the construct of identity. However, the most widely cited perspectives are ***biological, structural, sociocultural***, ***ecological,*** and ***post-structural***. The biological perspective associates identity to genes and their transmission from people to their children like their color, skin, and appearances. This view argues that one's identity is at least partially genetically transferred in a fixed way. The traditional structural perspective regards identity as a fixed and static attribute of an individual without taking contextual aspects into account [45]. Up against this view, the sociocultural approach considers identity as an active, dynamic, context-specific, and social practice [45]. Moreover, as pinpointed by Norton [46], the sociocultural perspective conceives identity as dynamic, complex, multiple, multifaceted, contradictory, (re)constructed through language, social-based, and transitory in relation to time and context. Another perspective inspired by the sociocultural perspective was the ecological approach that emphasized on the conceptual self or how people perceive themselves and how they think others think about them and their role in shaping their beliefs and the reverse [47]. The ecological stance also highlights the affordances and opportunities that a context may provide for an individual to act upon. This reminds us about the role of agency in identity construction and reconstruction [26].

Finally, the post-structural perspective regards identity as ongoing, shifting, multiple, dialogical, discontinuous, social, and multi-dimensional. The sociality element of this perspective is comparable to the sociocultural stance's proposition that identity grows out of social interactions [48]. According to the post-structural approach, identity is shaped and reshaped in relation to one's social and cultural context, beliefs, practices, norms, and discourses [39]. Drawing on these interpretations, it can be contended that sociocultural perspective breathes in the “discourse” of the post-structural approach to identity. While the post-structural perspective views teacher identities as constructed in discourse, sociocultural theories perceive identity as a social practice in a social context [4]. In sum, the multitude of perspectives on identity signifies that the construct is complicated and difficult to empirically measure. Hence, to capture the (re)construction and transition of teacher identity in EFL contexts, it is wise to benefit from a mixture of perspectives in scholarly attempts and interpretations.

#### 2.1.2 Teacher professional identity

As teacher identity positioned itself in the center of many recent explorations in teacher education, the concept of teachers' professional identity found an unprecedented interest among educational researchers in different fields. This led to numerous definitions and interpretations of the construct [11,49]. The reasons for this lack of unity in providing a solid definition for teacher professional identity concern its context-specificity and being shaped and reshaped outside a teacher. That is to say, in his/her environment, practices, and social interactions. Consequently, it can be argued that identity is a social variable that exists in social settings and in different forms involving sociocultural features. It is complex owing to the continuous interactions between the identities shaped in social contexts. In simple words, teachers' professional identity refers to how they perceive themselves as teachers based on their interpretations of constant interaction with their environment [11]. It also concerns a person's internalized expectancies concerning his/her professional role [50]. This sense arises from the ongoing interaction between the individual, context, and other peripheral characteristics and reveals itself in teachers' instructional performance, classroom practices, planning, satisfaction, commitment, self-efficacy, confidence, motivation, and more.

Despite a growing body of research on teachers' professional identity, most studies on this construct come under three research strands as pinpointed by Beijaard [11]. They reviewed studies in this area and argued that this line of research has chiefly focused on (a) teachers' professional identity formation and development, (b) finding features of teachers' professional identity, and (c) teachers' narrative stories of their professional identity. There are still many unexplored avenues in this area including the application of positive psychology to teacher identity [e.g., 51, 52] together with teachers' agency and positioning which can considerably affect teachers’ professional identity formation and reformation.

### 2.2 The definition of agency

The concept of agency has been given different definitions and descriptions by scholars in different fields. Simply, it is one's capacity and power to take actions, make decisions, and make an influence on his/her personal and professional life [16,22,53,54]. Other scholars perceive the concept differently and take related factors into account in their definitions of agency. For Ray [19] agency is a deliberate act to transform the person or his/her situations by his/her own actions. Likewise, Ollerhead [55] defined agency as one's capability to exercise choice and discretion in his/her daily practices. This construct is associated with and involves people's will, initiative, autonomy, self-regulation, freedom, volition, motivation, self-consciousness, deliberation, and choice [[56], [57], [58], [59], [60]]. Furthermore, agency is a socio-culturally mediated ability to act upon environment and practice that emanates from one's engagement in the social context [61,62]. It goes beyond simply taking initiative and is connected to circumstances wherein individuals take control of their life due to a perceived sense of duty [54].

#### 2.2.1 Teacher agency: definitions, conceptualizations, and dimensions

As a concept in its own right, teacher agency has not caught sufficient attention among educational scholars [22]. It concerns how teachers manifest their teaching practice and deal with school agendas and policies [21,63]. Moreover, Sahragard and Rasti [64] defined teachers' agency as the way they perceive and react to the shifting dynamics of their immediate educational context as well as local and global factors that affect their life and profession. Traditionally, it has been conceptualized as an individual attribute that concerns one's capability to influence actions and events. However, sociocultural and sociological traditions perceived teacher agency as a variable emerging from an interplay of personal and contextual factors. These perspectives highlight one's active participation, engagement, and mediation in social contexts [24]. In a similar manner, pragmatist approaches view agency as an offshoot of an interaction of personal abilities and contextual conditions. Hence, it is contended that teacher agency is achieved and affected by personal efforts, resources, and socio-structural factors. It is a complex construct that requires teachers to act by environment rather than simply act in the environment [65].

**Fig. 1:**
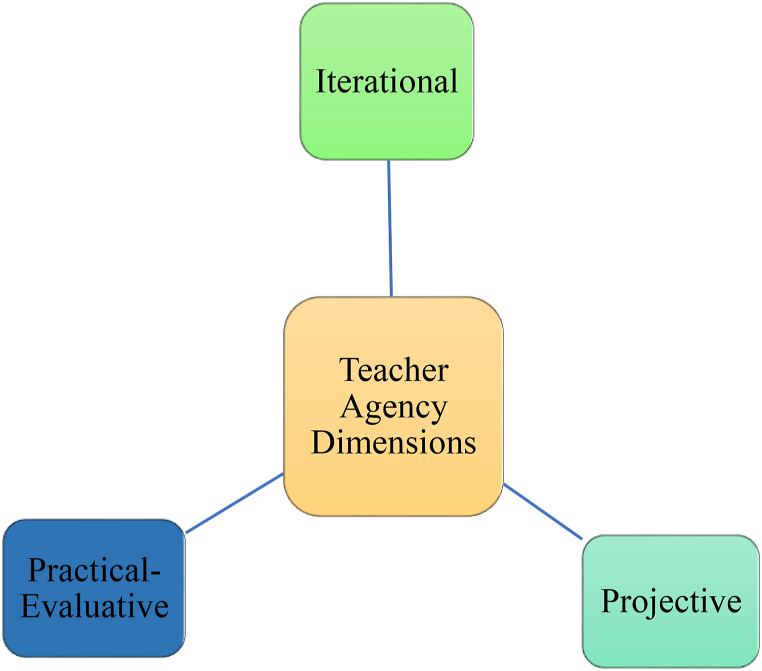
The dimensions of teacher agency.

As for teacher agency's dimensions, in their seminal study, Priestley et al. [24] took an ecological approach and regarded agency as a temporal-relational construct comprising of three dimensions: *iterational, projective,* and *practical-evaluative* (Fig. 1). The iterational dimension maintains that teacher agency is the by-product of activating accumulated past teaching experiences, patterns of thoughts and actions, and professional qualifications. These patterns permeate into teachers' classroom practices and make their identities stable. This dimension draws a demarcation between life experiences and career experiences whose amalgamation helps teachers in dilemmas and challenges at work. The projective dimension is future-oriented and includes one's short-term and long-term future aspirations which lead to his/her reconfiguration of actions in tune with future desires. Finally, the practical-evaluative dimension suggests the actor's ability to make conscious, practical, and normative judgments in a present situation while taking cultural, structural, social, and material situations into account. This sociocultural concern entails the dialogic nature of agency by which an actor interacts with other members of a community of actions and facilitates the achievement and sustainment of agency.

It is worth noting that these dimensions form a dynamic and complex relationship that varies from context to context. Hence, teacher agency must be seen as an interplay of various factors such as personal attempt, resources, organizational structure and climate, and contextual attributes. It is a configuration of past experiences, future aspirations, and present-time engagement in an action/event. This stresses the complexity of teacher agency as it requires many factors and things to materialize in his/her pedagogy.

### 2.3 Positioning theory

**Fig. 2:**
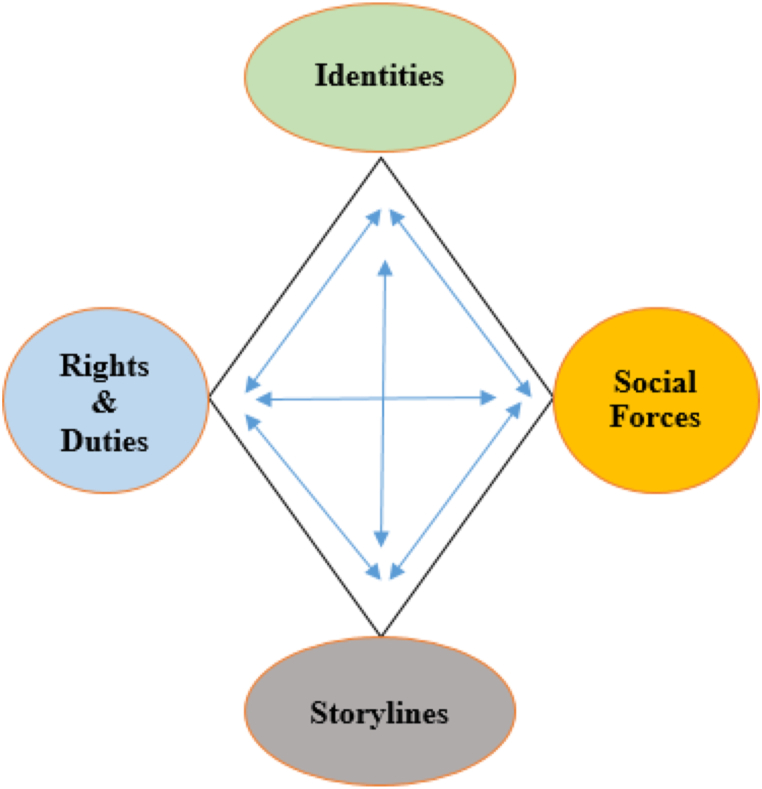
Positioning Theory Diamond [ [70], p. 92].

Positioning theory is an analytic tool to unpack how individuals see and perceive the world due to their roles in society. It was proposed by Harré and Davies [66] in social psychology to explain how people's positioning in their interactions with others affects their life experiences and identity development. It highlights the role of language and discourse in (re)shaping one's social reality and identity. In this theory, “role” differs from “position” in that the former is fixed and static, while the latter is dynamic and fluid [67]. Furthermore, positioning theory highlights context-specific actions and positions that are reflected in social interactions among members of groups, institutions, and cultures. The main purpose of this theory is to investigate the order of interlocutors' social actions, rights, and duties by which they act and speak in certain ways. Therefore, it capitalizes on the social domain in which these rights and duties are distributed, negotiated, and disputed [68]. In this theory, positioning depends on three aspects of interaction namely, storyline (i.e., interactions), speech acts, and assigned/adopted positions [69]. According to this tripartite, any speech act can be self-positioning or other-positioning. Furthermore, trying to develop a systematic and *trans*-disciplinary framework for examining discourse and positioning, Slocum‐Bradley [70] proposed a diamond model for positioning which included rights and duties, identities, social forces, and storylines (Fig. 2).

Spreading its seeds in other fields (psychology, journalism, public relations), positioning theory found its way to education and applied linguistics over the last decade [27]. It has been utilized to understand classroom interactions and the ways it affects students' language learning and identity formation process. Moreover, this theory has been used to examine how students position themselves or are positioned in educational programs. Likewise, teachers’ positing has been recently studied focusing on how teachers position themselves and their students in classroom context/interactions [27]. Many studies on positioning theory and teachers have focused on their identity construction [25]. This theory argues that teachers basically reposition their identities in relation to their classroom practices, roles, and organizational culture [28]. It is still a fresh theory in language education that has numerous potentialities to help teachers and researchers understand from classroom interactions, experiences, and behaviors [27].

#### 2.3.1 Modes and dimensions of positioning

Two modes have been proposed for the concept of positioning in the available literature including reflexive positioning and interactive positioning. As stated by Davies and Here [66], reflexive positioning is the positioning of self and the expression of personal identity. On the other hand, interactive positioning is other-oriented and is defined simply as the positioning of others in a joint interaction. Both modes can be intentional or unintentional depending on the aim the individual has in mind. As put by Kotsopoulos [71], interactive positioning can be challenged by another person's reflexive positioning. It may happen by one person toward another or as a joint action by a group of people. It is essential to note that both modes are transferable and mutable across contexts and times [72].

Positioning has two main dimensions namely, *position order* and *intentionality*. Position order is explained as the instance by which a position is specified. It can be an initial position, a reposition, or even a position transferred from another context. However, position intentionality concerns how purposefully a position is assumed and whether it is conducted as an action to affect others or as a fulfillment of others’ expectations [73].

### 2.4 Empirical studies

Positioning has been illuminating in the areas of teachers' identity and agency over the past years [6]. As Tran and Pasura [74] stated, teacher positionings (reflective and interactive) facilitates professionalism and productive interactional relationships with students that develops their L2 socialization [75]. Both identity and agency are approved to be dynamic, multiple, social, and interaction-based. Their construction and reconstruction is a function of an interplay of internal and external factors. In some cases, one's identity and agency are challenged or contested by self and others [76]. This highlights the importance of positioning and the way a teacher is perceived by him/herself and others in a social milieu [29]. Teachers' positioning is a relational concept in that teachers usually position themselves in relation to others (e.g., students, supervisors, parents, colleagues, institutions). Hence, the manner by which a language teacher sees and positions him/herself in interactions with others meaningfully contributes to his/her identity (re)construction and development [77]. In their recent longitudinal study, Mehdizadeh et al. [78] examined a single teacher's professional identity development and repositioning using interviews and observations and found that the participant's identity and positioning changed given the surrounding community of practice and evolving professional context. Likewise, Mansouri [41] explored two EFL teachers in Iran to reveal their identity positionings as reflected in their lived experiences. Using interviews and journals, they found that each teacher that each teacher took different approaches in positioning her identity in their storylines.

Moreover, in a qualitative case study [1], examined three ESL teachers' identity (re)negotiations and agency in the United States and found that the participants took different, and sometimes conflicting, positional identities with respect to their social context that shaped their agency and classroom performance. Likewise, in the context of Australia, Turner [79] explored two teachers' positioning acts and their effect on their agency and pedagogical practices. The results indicated that teachers' ways of positioning in the classroom strongly shape their agency and practice in the classroom. This echoes what Tait-McCutcheon and Loveridge [80] put in their seminal research which demonstrated that teachers’ positioning of self and students in the class have more power than their classroom materials and resources.

Moreover, as the classroom is a mini social setting where interactions frequently occur, teachers' self-positioning and other-positioning behaviors, directly and indirectly, influences students' identity and agency as well [28]. At a wider level, the ways EFL teachers assign themselves and their students' specific positions stop or facilitate their classroom engagement, learning, communication, and acquisition [[80], [81], [79]]. The growing body of knowledge in this line of research substantiates that teachers' positioning, professional identity, and agency have a robust relationship that is complicated and mutually established [82,83]. In Finland, Green and Pappa [84] argued that EFL teachers' professional agency has several manifestations in teacher educators' decisions and practices. Taking a poststructural perspective, Nazari and De Costa [85], investigated the role of institutional power in shaping Iranian EFL teachers' autonomy, agency, and identity. They utilized narrative frames and semi-structured interviews to gather their data. The results showed that the power bore in academic centers determines teachers' practice, autonomy, agency, and identity as teachers. Similarly, in a longitudinal study, Wu [86] examined a Chinese EFL teacher's agency and sustainable identity growth in light of positioning theory through narratives and interviews. In the end, it was found that consistent teacher agency and positioning foster professional identity (re)negotiation. Moreover, they endorsed the role of teacher emotions and emotion-regulation strategies in shaping agency.

In light of these seminal investigations, therefore, it is tenable to claim that people do not act in a certain way unless they realize an identity position for themselves from that act. These studies signify that researching this scholarly domain is by no means totally uncharted in applied linguistics, yet it requires more complementary studies in EFL contexts and in association to other variables, especially those related to teachers. Against this lacuna, the current review article provided a thorough account of theoretical and empirical underpinnings of three booming constructs in language education namely, teachers’ positioning, professional identity, and agency.

## 3 Concluding remarks

In light of this theoretical review, it was maintained that EFL teachers positioning, professional identity, and agency are all socially constructed and arise from interactions that teachers have in the classroom and organizations. Many internal and external factors were claimed to affect these variables and their relationship. Moreover, the ways teachers and students interact and perceive themselves and others were found to affect who they are and how they act agentively in the class. This signified the prominent role of language and discourse in the (re)construction and (re)negotiation of teachers’ professional identities and agency in EFL contexts. Other than theoretical benefits, this article has practical implications for different stakeholders in EFL contexts as well. For instance, EFL teachers can use the ideas and propositions made in this study to upsurge and raise their knowledge and consciousness about the role of interactional discourse and positing self and others in classroom conversation in developing their identity as teachers and their agentic power to take initiatives and act in a specific way in an educational context. Moreover, EFL teachers can identify the complexity and dynamism of their positioning, professional identity, and agency which are all affected by numerous factors inside and outside the person. In a similar vein, EFL students can benefit from the findings of this study in that they can understand the impact of positioning on their language learning, classroom participation, motivation, agency, and identity as learners. The way they position themselves and are positioned by teachers in interactions strongly affects their language development.

The review is also valuable for EFL teacher educators in the sense that they can run workshops and training courses in which the role of positioning strategies and practices in shaping different aspects of teacher education are highlighted. In such training courses, teacher educators can offer practices by which different forms of teacher identities, positioning, and agency are taught in relation to various contextual peculiarities. The trainers can also elaborate on different theories and models regarding these variables and ask teachers suggest practical strategies depending on their own experiences and classes. They can also work on developing EFL teachers' interactional aspects like positioning self and others in a conversation in addition to pedagogical/instructional techniques that they inculcate in pre-service and in-service teachers. Another group to take advantage of the propositions of this study relates to materials developers who can develop textbooks, tasks, and activities that reflect the dialogical nature of positioning, agency, and identity development. They can revisit tasks and activities to tap into teachers' and students' agency and positioning and their transitions from task to task. Furthermore, the study is helpful for school principals and administrators in that they can be aware that how they position the teachers in the institute can affect their identity, agency, autonomy, job commitment, and classroom performance. Hence, they try to establish a friendly interactional discourse with EFL teachers in order to bring about positive outcomes to education. Additionally, the present study might have pedagogical implications for EFL teachers' professional development in that they may realize the criticality of agentic behaviors in shaping their identity and pedagogical efficacy. In light of this research, EFL teachers’ professional development programs may shift from mere instructional aspects toward agency and positioning as the underlying elements of their identity and professionalism.

Finally, L2 researchers can use this study and run similar investigations and bridge the yawning gaps in this line of inquiry across various contexts. This conceptual study can boil down to empirical studies on how different community of practices can foster EFL teachers' agency, positioning, and identity (re)construction using positioning theory, poststructural, sociological perspectives to analyze data. The dialogic nature of each of the variables can also be explored by future scholars. As stated earlier, most of the studies conducted on this strand of research have been carried out in Anglophone countries like the United States and Australia and non-native English speaking countries where English is regarded as EFL have been ignored. Hence, avid scholars can run comparable studies on EFL teachers' positioning, professional identity, and agency. Likewise, cross-cultural studies in this domain are absent despite the fact that the three constructs of positioning, professional identity, and agency are context-specific. Consequently, future studies can compare different cultures and how they perceive these variables and their outcomes to language education. Another gap in this area is that the majority of studies on the variables mentioned have focused on students' perspectives, while teachers and supervisors' views have limitedly caught scholarly attention in EFL contexts. So, how administrators and supervisors position themselves in relation to teachers and vice-versa is a novel idea for research. Moreover, the existing tensions and conflicts between these parties that naturally affect their positioning, identity, and agency can be an area for further research. Novice teachers are usually fraught with tensions in their early years of teaching which in some cases culminate in leaving the job [87]. These conflicts considerably affect teachers’ identity formation process as they wish to be autonomous agents of their own practice, while schools and organizations have a different vision [88].

Additionally, reflexive and interactive positioning can be explored in association with teacher-student interpersonal communication factors such as immediacy, credibility, clarity, and the like due to the interactional nature of positioning [e.g. Ref. [89]]. Similarly, future studies can be conducted using positive psychology perspective in examining teacher identity [e.g. Ref. [90]]. Another direction can be conducting qualitative, longitudinal, and even mixed-methods research studies on the variables of concern in this article as the existing studies are mostly case studies with limited generalizability scope. To capture the dynamics and developmental trajectories of positioning, professional identity, and agency of EFL teachers, it is wise to use narrative experiences, diaries, portfolios, classroom observations, and in-depth interviews as data collection instruments in future studies. Qualitative studies on the factors that influence these constructs are also suggested. The role of agency, identity, and positioning in L2 assessment, especially across various testing conditions and language skills can also be the subject of further research [91,92]. More specifically, the positioning and agency of EFL teachers in light of alternative assessments (e.g., learning-oriented assessment (LOA) and the way they influence their identity is an uncharted domain for research in the future [[93], [94], [95]].

Last but not least, in online education and cyberspace, EFL teachers usually take on different positioning acts, identities, and agentic behaviors. Hence, it is insightful to carry out similar studies in the context of e-education to see if these variables go through changes or not as a result of technologies and context shift [96]. All these points demonstrate that despite its growing trend, researching EFL teachers’ positioning, professional identity, and agency still has many unexplored avenues for research and calls for attention from scholars all around the globe.

## Author contribution statement

All authors listed have significantly contributed to the development and the writing of this article.

## Data availability statement

No data was used for the research described in the article.

## Additional information

No additional information is available for this paper.

## Declaration of competing interest

The authors declare that they have no known competing financial interests or personal relationships that could have appeared to influence the work reported in this paper.
